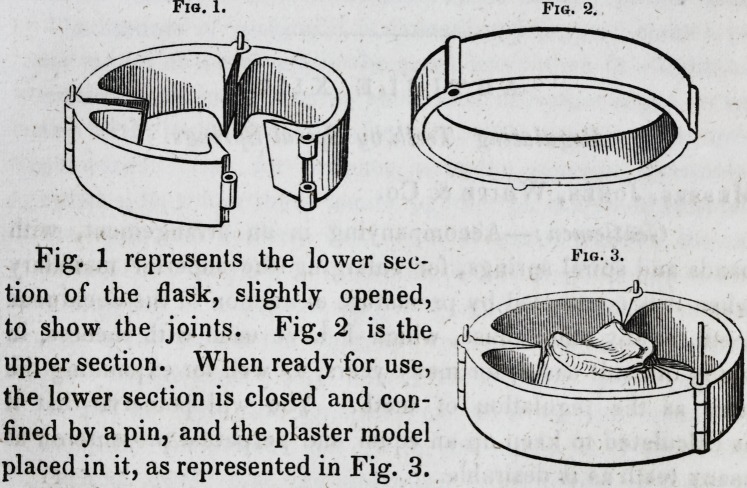# Hawes' Moulding Flask

**Published:** 1850-10

**Authors:** 


### SELECTED ARTICLES.

### ARTICLE XII.

### *Hawes' Moulding Flask*

At the last annual meeting of the Society of Dental Surgeons of the State of New York, Mr. George E. Hawes, of this city, exhibited, in connection with his experiments on metallic casts, a new flask for moulding models, which, owing to the depression of the jaw above the most prominent portion of the gums, cannot be removed perpendicularly from the simple flask, in common use, without dragging more or less sand with it. This "drag" prevents the dentist from procuring a perfect casting, which is ensured in all cases by the use of Mr. Hawes' new flask.

The following cuts will illustrate the operation of this flask with very little description:

Fig. 1.

Fig. 2.

Fig. 3.

Fig. 1 represents the lower section of the flask, slightly opened, to show the joints. Fig. 2 is the upper section. When ready for use, the lower section is closed and confined by a pin, and the plaster model placed in it, as represented in Fig. 3.

If the model be considerably smaller than the space between the flanges, projecting in towards it, small slips of paper may be placed in the joint extending to the sides of the model, to part the sand when opening the flask for the removal of the pattern. The sand may now be tamped around the pattern up to the most prominent part of the gum, and it should be finished smoothly around it, slightly descending towards the model, so as to form a thick edge of sand for the more perfect parting of the flask. The sand and face of the model must now be covered with dry pulverized charcoal, sifted evenly over the whole surface. The moulders keep it in a bag which they shake over the flask.

When this is done, the upper section of the flask is placed upon the lower, and carefully filled with sand. It is then raised from the lower one, which may then be parted, by removing the long pin, and the model gently taken away. When closed, and the two put together again and inverted, it is ready to receive the melted metal.

We have used this flask, for which we are indebted to Mr. Hawes, for some months; and have been able to make, by its use, more perfect castings than ever before, in the kind of cases for which it was designed.

We understand that Mr. Chevalier will soon have an assortment of them for sale.—*N. Y. Dent. Rec.*

## Figures and Tables

**Figure f1:**